# Influence of epidemic situation on COVID-19 vaccination between urban and rural residents in China-Vietnam border area: A cross-sectional survey

**DOI:** 10.1371/journal.pone.0270345

**Published:** 2022-07-21

**Authors:** Bin Liu, Min Zhang, Xiangang Li, Li Liu, Qin Li, Zhengzhong Liang, Lin Xu, Li Li, Yuekang Su

**Affiliations:** The Fifth Affiliated Hospital of Kunming Medical University, Diannan Central Hospital of Honghe Prefecture (Gejiu People’s Hospital), Gejiu, Yunnan, China; The Chinese University of Hong Kong, HONG KONG

## Abstract

**Background:**

The situation of the COVID-19 outbreak in the border areas of China and Vietnam is complex, and its progress may affect the willingness of urban and rural residents to receive the vaccine.

**Objective:**

This study aims to understand the influence of the COVID-19 epidemic situation on the willingness of urban and rural residents in China-Vietnam border areas to get vaccinated and the factors that affect the vaccinations.

**Methods:**

A cross-sectional survey was conducted in Hani-Yi Autonomous Prefecture of Honghe, a border area between China and Vietnam, using online and paper questionnaires from April 1 to June 4, 2021. A total of 8849 valid questionnaires were surveyed to compare the differences in the willingness of urban and rural residents to receive the COVID-19 vaccine. Single factor analysis and multivariate logistic regression analysis were used to explore the influence of the epidemic situation on the willingness to be vaccinated.

**Results:**

In the border areas between China and Vietnam in Yunnan Province, both urban and rural residents had a high willingness (> 90%) to receive the COVID-19 vaccination, with a higher level of willingness in urban than in rural areas and a higher willingness among residents aged ≥ 56 years. Rural residents mainly concerned about the vaccination were different from urban residents (*p*< 0.05). About 54.8% of urban respondents and 59.2% of rural respondents indicated that their willingness to get COVID-19 vaccine would be affected by new COVID-19 cases. Respondents who were divorced, had an occupation other than farming, had contraindications to vaccination, were concerned about the safety of vaccines and worried about virus mutation, thought that the epidemic situation would not affect their willingness to get vaccinated (*p*< 0.05).

**Conclusion:**

The prevention and control of epidemics in border areas is of considerable importance. It is necessary to conduct targeted health education and vaccine knowledge popularization among urban and rural residents to increase the vaccination rate and consolidate the epidemic prevention and control at the border.

## Introduction

Coronavirus Disease 2019 (COVID-19) is an acute infectious disease, and the worldwide outbreak thereof is a serious threat to people’s lives and health [1]. As of August 16, 2021, more than 207 million people in over 200 countries and regions had been infected and 4.36 million had died [2]. According to the regional division of the World Health Organization (WHO), the cumulative number of confirmed cases in Southeast Asia [3] accounts for one quarter, exceeding 39.99 million, with over 0.6 million deaths. In Asia, India currently faces the most severe situation for COVID-19 epidemic prevention and control [4], and the B.1.617 variant was discovered there. For epidemic prevention and control, variant strains have become a new challenge because they can circumvent immunity and lead to increased infectivity.

Vaccination against COVID-19 is the most economical and effective means to control the COVID-19 epidemic [5]. Studies have shown that the new coronavirus may coexist with humans for a long time [6], and that new viruses and new mutant [7] strains may emerge. Only by vaccination as soon as possible to form an effective immune barrier [8] can the epidemic be controlled. With the Progress of the epidemic situation, vaccination against COVID-19 may be a long-term, ongoing effort. The development of the epidemic situation at the border may affect the willingness of the public to be vaccinated. There may be differences in willingness and perception of vaccination against COVID-19 between urban and rural residents of the border due to differences in economic base, education level, and medical security [9].

China, the world’s most populous country [10], was one of the first countries to effectively respond to the COVID-19 outbreak due to the strict and effective outbreak prevention and control strategies [11, 12] adopted by the government at the beginning of the outbreak. However, China is vast and shares borders with 14 neighboring countries, including India, Russia, Myanmar, and Vietnam. The total length of the border is over 55,000 kilometers [13]. Neighboring countries such as India and Vietnam have experienced continuously worsening domestic outbreaks of COVID-19 [14]. The effective control of the COVID-19 epidemic in border areas has become the sticking point to national epidemic prevention and control.

Yunnan Province is one of the provinces with the richest tourism [15] resources in China’s southwestern border areas, bordering Myanmar, Vietnam, Laos, and other Southeast Asian countries. Due to the long border line, close border personnel exchanges, and difficult border epidemic prevention and control [16], the border of Yunnan has become a key province for the prevention and control of imported infectious diseases in China. Hani-Yi Autonomous Prefecture of Honghe is located between 101°47′~104゜16′east longitude and 22゜26′~24゜45′north latitude [17]. It borders two provinces, one city and six counties in Vietnam, with a borderline of 848 kilometers, and there are two national first-class border ports, Hekou and Jinshuihe. Due to its unique geographical location, complex mountainous terrain, and the vast majority of residents living in mountainous areas, border residents’ perceptions of epidemic prevention and control and their willingness to receive COVID-19 vaccination may be different from others.

In summary, the willingness of urban and rural residents in China-Vietnam border areas to be vaccinated against COVID-19 is still unclear. Will the progress of the epidemic situation in the border areas affect their willingness to be vaccinated? What factors might change their willingness to be vaccinated against COVID-19? These issues require further research. Therefore, it is of great significance to explore the factors that affect the vaccination of border residents, especially which factors can be adjusted, in order to improve the vaccination rate and effectively curb the occurrence and spread of epidemics at borders.

## Materials and methods

### Research object

In China, COVID-19 vaccination is voluntary and free of charge for Chinese citizens, and they can choose the nearest vaccination site. Urban and rural residents along the China-Vietnam border in Hani-Yi Autonomous Prefecture of Honghe, Yunnan Province, China, were selected as the survey respondents. Participation in this COVID-19 vaccination-related questionnaire is at the discretion of the respondents and is completely voluntary.

### Calculation of sample size

According to previous research [11] and preliminary investigations on the cognition of the epidemic situation in COVID-19 and the vaccination willingness of ordinary adult residents in Hani-Yi Autonomous Prefecture of Honghe, those who had a negative attitude towards the epidemic situation accounted for 28.5% (p≈0.285) of the population, with the allowable error δ = 5% and the inspection level α = 0.05, 1-α = 0.95. Considering the loss of follow-up rate of 15%, the sample size for the cross-sectional study calculated by software PASS 15.0.5 was n = 4511 cases. In order to ensure that the present study was more representative, the sample size was calculated as 9022 cases.

### Compilation and improvement of the questionnaire

According to the epidemic situation in neighboring countries, we interviewed the local urban and rural residents about their awareness of and willingness to receive vaccination in COVID-19, and prepared the questionnaire on awareness of the epidemic situation and willingness to receive vaccination in COVID-19. It includes a self-assessment of one’s own health, willingness to be vaccinated, factors affecting vaccination, the impact of the border epidemic situation on the willingness to get vaccinated and other related issues. The questionnaire was continuously optimized and perfected based on the potential problems in the epidemic prevention and control process. Given that some respondents might not have or know how to use a smartphone, a paper questionnaire with the same content as the electronic was prepared. They can choose either one to participate in the survey, and each person can only participate once.

In the early stage of COVID-19 vaccination promotion, residents may have doubts about their eligibility for COVID-19 vaccination, so during the questionnaire survey, respondents were asked to make a self-assessment of their health status [18]. Evaluation criteria for physical health status were based on the WHO definition of health [19]. Health (YES): no acute or chronic diseases, physical and mental health; non-Health (NO): sub-health and disease state, sub-health: no physical disease, but with high pressure in physiological, psychological and social states, disease status: acute and chronic diseases and/or long-term medication. During the pre-vaccination self-assessment of health and the formal vaccination, medical staff check indications and contraindications, ask questions about the history of acute and chronic diseases and sign an informed consent form prior to the formal vaccination through a preliminary medical examination.

#### Survey content

The questionnaire included general information (gender, age, marital status, education, occupation, work/income status), personal knowledge of local epidemic prevention and control, and the relationship between the epidemic situation of neighboring countries and the willingness of urban and rural residents to get vaccinated against COVID-19.

### Evaluation and quality control of the questionnaire

After the questionnaire was compiled, the content, structure, and logic of the questionnaire were continuously improved and perfected to make each question of the questionnaire more representative, and epidemiological experts were invited to conduct a quality assessment and feasibility analysis of the questionnaire. Before the formal questionnaire survey, the designed electronic questionnaire was pushed to the class/department/family group through the WeChat of the mobile phone, and voluntary participants were invited to complete the initial test of the questionnaire. A total of 177 electronic questionnaires were collected. The option of "degree" evaluation was used for content consistency evaluation. The Cronbach alpha value was 0.825, and the mean inter-item correlation was 0.612. The accuracy of the responses for gender, age, occupation, household registration and education level, etc. were evaluated one week after the initial assessment. A total of 105 participants completed the two evaluations. The Pearson correlation coefficient of the two tests was calculated, and the correlation was 0.926, *p*<0.01, indicating that the test-retest reliability of the basic information of the questionnaire is good, and the stability is high.

### Implementation of the investigation

The sample size required for the survey is determined by the proportion of the population in each county, and the number of people surveyed in each area is allocated based on the population size. Digital random sampling was used to select corresponds. The city of Gejiu and the county of Hekou County, Jinping County, Luchun County, and Yuanyang County were selected from 13 counties and cities. Based on the proportion of the population, there were five natural villages and two urban communities in 1 township that were randomly selected from county-level cities. One urban community and 1-to 2 natural villages were randomly selected from 4 counties. Before the investigation, the investigators had to be uniformly trained to make the investigation results homogeneous. Participates should carefully read the notice of voluntary and anonymous participation on the homepage of the questionnaire. When they choose the option to agree to participate, they can be continual to complete the questionnaire, otherwise they can withdraw at any time. Incomplete questionnaires will be invalid and will not be recovered. The survey was conducted on-site from April 1 to June 4, 2021, with informed consent and voluntary participation, and was conducted through "Questionnaire Star" (https://www.wjx.cn). Electronic questionnaires were mainly used, but paper questionnaires were available for elderly people, people without smartphones and people who did not know how to submit the questionnaire online. The electronic questionnaire was filled in by scanning a QR code on the mobile phone app WeChat and logging in to "Questionnaire Star". The paper questionnaire was filled out by the on-site investigator during on-site follow-up. Each person was restricted to fill in one electronic questionnaire per mobile phone, or to fill in one paper questionnaire. One could not complete the electronic questionnaire and then also complete the paper questionnaire, and one could not complete the electronic questionnaire repeatedly. Each mobile phone number was restricted to fill in one electronic questionnaire. All options of the electronic questionnaire were set as mandatory options, and the questionnaire could only be submitted after all the options were answered.

### Ethics

The protocol of the current study was reviewed and approved by The Fifth Affiliated Hospital of Kunming Medical University, and Ethics Committee approved No. AF-SQ-2021-019.

### Statistical analysis of data

Statistical analyses were conducted using the Statistical Package for Social Sciences, SPSS, SPSS24.0 (IBM). The enumeration data were described by frequency and percentage, and the *χ2* test was used for comparison between groups. The factors of basic personal information, self-evaluation of residents’ health status, willingness to vaccinate and self-awareness to vaccinate against COVID-19 were analyzed with the single logistic model. All possible potential related factors with *p*<0.25 [20] in single factor analysis were in multivariate logistic analysis, the test level α = 0.05, use logistic Enter, and the ratio (*OR*) and 95% confidence interval (95% *CI*) were calculated. The analysis of residents’ willingness to get vaccinated against COVID-19 in urban and rural areas was expressed as the frequency composition ratio of the population of each age group (%).

## Results

### Basic information of the respondents

A total of 9063 respondents participated in this questionnaire survey, of which 95% were online. All online questionnaires collected were complete, but 45 paper-based questionnaires with missing items were considered as unqualified. 214 of the questionnaires were considered unqualified due to the short response time, age under 18 and contradictory options. There were 8849 valid questionnaires, and the effective recovery rate was 97.6%, of which 41.3% were completed by males (3657) and 58.7% by females (5192). The ages of the respondents were mainly 18–55 (88.4%), and 68.9% of the respondents were married. In urban areas, 63.3% of survey respondents had a college degree or above, and 85.5% said that they were wealthy/had guaranteed income. Meanwhile, rural residents were mainly engaged in farming (57.8%), and most of said residents had an education level of primary school or below (58.8%). In rural areas, 71.3% of respondents indicated that their income was guaranteed (Table 1).

**Table 1:** Basic characteristics of the surveyed urban and rural population (n = 8849).

Demographic		Urban n	%	Rural n	%	Total n	%
Gender	Male	1914	36.7%	1743	47.9%	3657	41.3%
	Female	3296	63.3%	1896	52.1%	5192	58.7%
Age(years)	18–35	2353	45.2%	1617	44.4%	3970	44.9%
	36–55	2250	43.2%	1597	43.9%	3847	43.5%
	≥56	607	11.7%	425	11.7%	1032	11.7%
Marital status	Unmarried	1322	25.4%	869	23.9%	2191	24.8%
	Married	3521	67.6%	2580	70.9%	6101	68.9%
	Divorced	312	6.0%	129	3.5%	441	5.0%
	Widowed	55	1.1%	61	1.7%	116	1.3%
Education	Primary school or below	849	16.3%	2141	58.8%	2990	33.8%
	Secondary/high school degree	1062	20.4%	733	20.1%	1795	20.3%
	College/Undergraduate	3253	62.4%	760	20.9%	4013	45.3%
	Master’s degree or above	46	0.9%	5	0.1%	51	0.6%
Profession	Public officials	1268	24.3%	336	9.2%	1604	18.1%
	Worker/self-employed	823	15.8%	310	8.5%	1133	12.8%
	Medical staff	1601	30.7%	370	10.2%	1971	22.3%
	Farmer	508	9.8%	2103	57.8%	2611	29.5%
	Other	1010	19.4%	520	14.3%	1530	17.3%
Job-status/income status	Stable job and wealthy	3421	65.7%	903	24.8%	4324	48.9%
	Unstable job but guaranteed income	1029	19.8%	1692	46.5%	2721	30.7%
	No fixed-job, basic income guarantee	345	6.6%	612	16.8%	957	10.8%
	No job, basic life difficulties	89	1.7%	190	5.2%	279	3.2%
	Students in school, no income	326	6.3%	242	6.7%	568	6.4%

### Comparison of urban-rural differences in related factors affecting COVID-19 vaccination

According to the survey results (Table 2), there were significant differences between urban and rural areas in terms of residents’ health status, contraindications to vaccination, concerns about virus mutation, and concerns about payment for the COVID-19 vaccination (*p*< 0.001). For rural residents, the proportion of people in poor health, with contraindications for vaccination and worried about whether or not to pay for the vaccine was higher than that of urban residents. Meanwhile, urban residents were more worried about the safety of the vaccine and virus mutation.

**Table 2:** Comparison of urban-rural differences in related factors affecting COVID-19 vaccination (n = 8849).

Factors		Urban n(%)	Rural n(%)	Total n(%)	χ^2^	*p*
Self-assessment of own health	Yes (healthy)	3336(64.0%)	2222(61.1%)	5558(62.8%)	8.090	0.004
	No (not healthy)	1874(36.0%)	1417(38.9%)	3291(37.2%)		
Worried about the safety of vaccines	Yes	1514(29.1%)	1018(28.0%)	2532(28.6%)	1.234	0.267
	No	3696(70.9%)	2621(72.0%)	6317(71.4%)		
There are contraindications to vaccination	Yes	1194(22.9%)	957(26.3%)	2151(24.3%)	13.311	<0.001
	No	4016(77.1%)	2682(73.7%)	6698(75.7%)		
Concerns about virus mutation	Yes	858(16.5%)	468(12.9%)	1326(15.0%)	21.889	<0.001
	No	4352(83.5%)	3171(87.1%	7523(85.0%)		
Other factors	Yes	297(5.7%)	190(5.2%)	487(5.5%)	0.947	0.331
	No	4913(94.3%	3449(94.8%)	8362(94.5%)		
The impact on vaccination if the COVID-19 vaccine needs to be paid for	Whether the vaccine needs to be paid for or not has no affect	3502(67.2%)	2087(57.4%)	5589(63.2%)	167.897	<0.001
	Payment may affect	1059(20.3%)	974(26.8%)	2033(23.0%)		
	Very worried about payment and hesitating to get vaccinated	146(2.8%)	266(7.3%)	412(4.7%)		
	No vaccination if the vaccine needs to be paid for	503 (9.7%)	312 (9.7%)	815(8.6%)		

### Awareness of COVID-19 vaccination among urban and rural residents at the border

**Fig 1:**
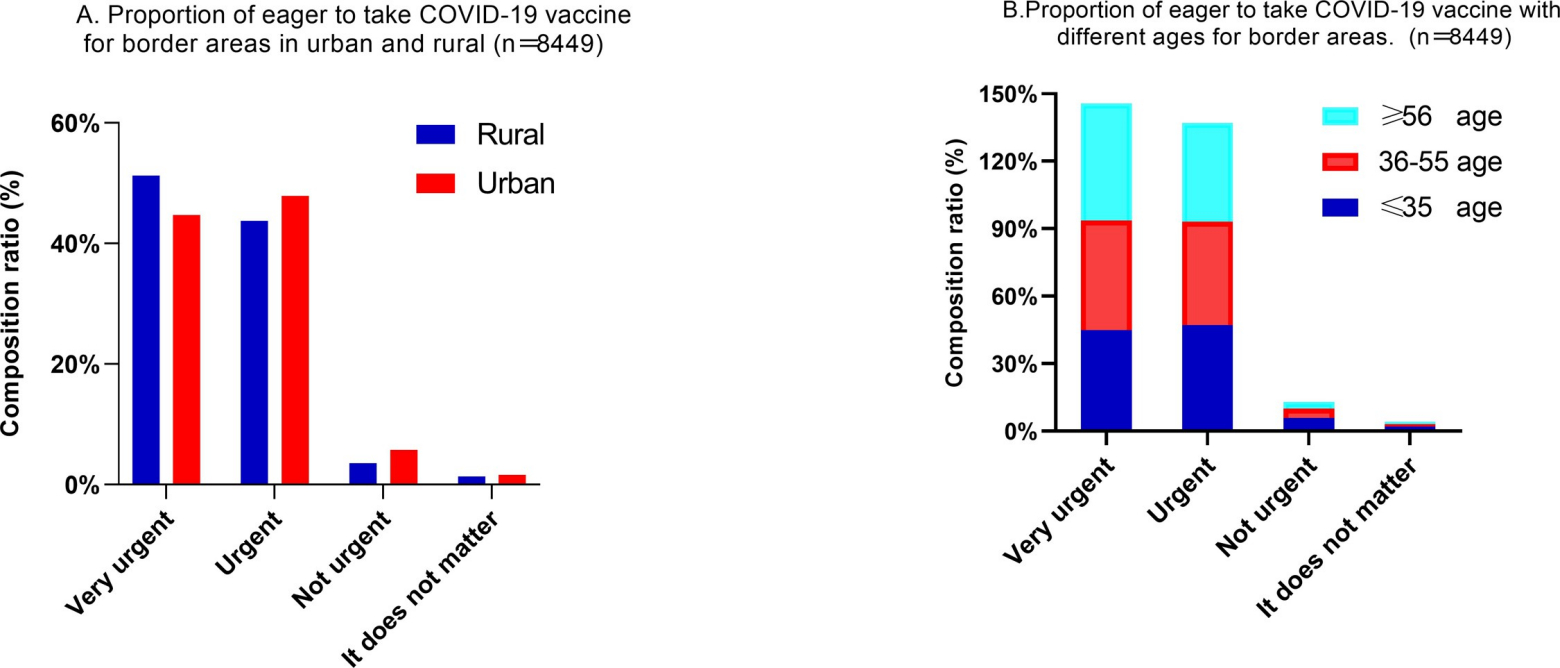
A) Border rural residents: 95.1% (3461/3639) urgently expecting COVID-19 vaccination, higher than the 92.6% (4826/5210) for urban residents. Especially, 51.3% (1866/3639) of rural residents show the highest demand. B) Those of age ≥ 56 have the strongest willingness, accounting for 52.0% (537/1032).

The results of the survey reveal that 94.7% of the border residents thought that the vaccine could effectively prevent and control the epidemic situation of infectious diseases, and 93.6% of border residents hoped to get the vaccine as soon as possible, and the percentage in rural areas was higher than that in urban areas (Fig 1A), especially those aged ≥ 56 years old (Fig 1B). The difference between urban and rural areas was statistically significant (*p*< 0.001). Over 95% of the border residents thought that the domestic COVID-19 vaccine was very safe or relatively safe, and more than 97% of the border residents said that they would take the initiative to publicize and mobilize others to get vaccinated (Table 3).

**Table 3:** Comparison of the differences in cognition of COVID-19 vaccination among urban and rural residents at the border (n = 8849).

		Urban n (%)	Rural n (%)	Total n (%)	χ^2^	*p*
Vaccination can effectively prevent and control the COVID-19	Definitely can	2643 (50.7%)	1747(48.0%)	4390(49.6%)	27.253	<0.001
	Should be able to	2332 (44.8%)	1659(45.6%)	3991(45.1%)		
	Won’t be able to	187(3.6%)	157(4.3%)	344(3.9%)		
	Do not know	48(0.9%)	76(2.1%)	124(1.4%)		
Eager to get the vaccine as soon as possible	Very urgent	2329(44.7%)	1866(51.3%)	4195(47.4%)	50.358	<0.001
	Urgent	2497 (47.9%)	1595(43.8%)	4092(46.2%)		
	Not urgent	300(5.8%)	128(3.5%)	428(4.8%)		
	It does not matter	84(1.6%)	50(1.4%)	134(1.5%)		
Safety of domestic COVID-19 vaccines	Very safe and assured	2842 (54. 5%)	1818(50.0%)	4660(52.7%)	33.970	<0.001
	Moderately safe	2321 (44.5%)	1748(48.0%)	4069(46.0%)		
	Not very safe	32 (0.7%)	54(1.5%)	86(1.0%)		
	Especially worried	15 (0.3%)	19(0.5%)	34(0.4%)		
Willingness to mobilize others to vaccinate	Yes	4280 (82.1%	2658(73.0%)	6938(78.4%)	105.084	<0.001
	Maybe	853(16.4%)	901(24.8%)	1754(19.8%)		
	No	54(1.0%)	58(1.6%)	112(1.3%)		
	Nothing to do with others	23(0.4%)	22(0.6%)	45(0.5%)		

### The impact of new COVID-19 cases on the willingness of urban and rural residents to be vaccinated against COVID-19

**Fig 2:**
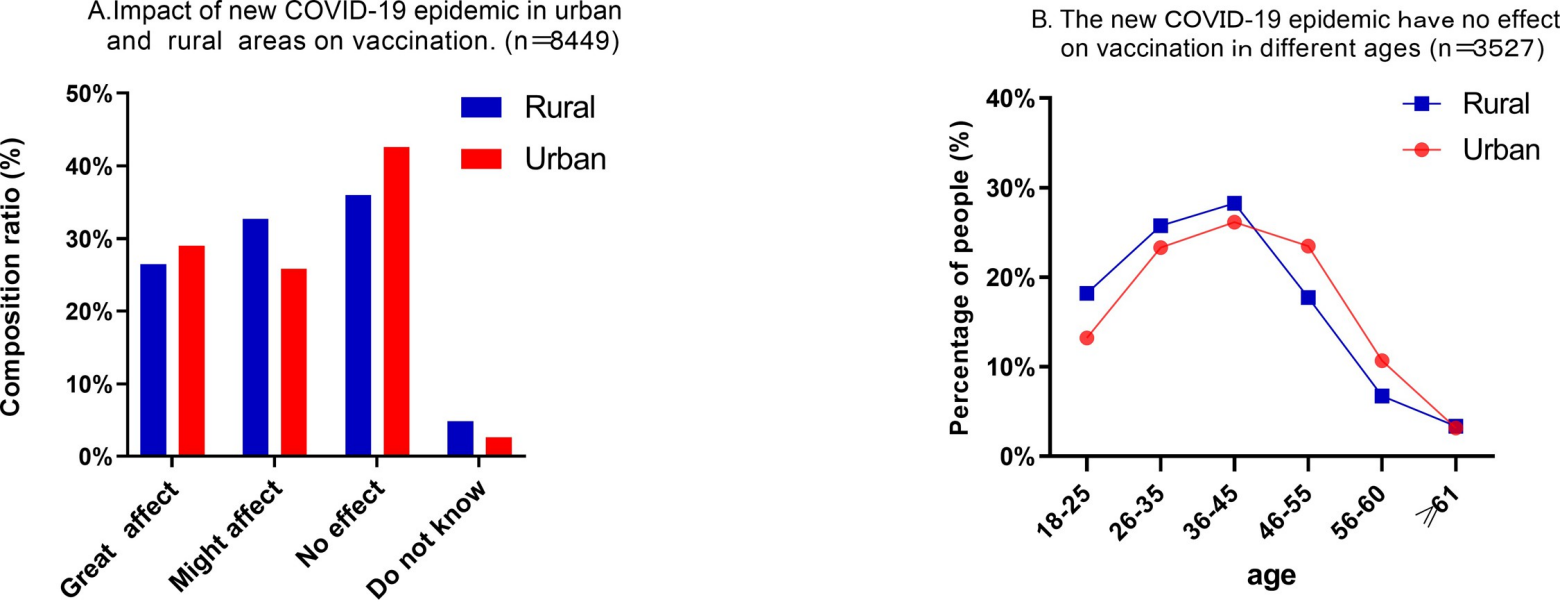
A) Rural areas: 59.2% (2154/3639) thinks so, higher than the 54.9% (2855/5210) for urban areas. B) border residents of age ≤45 believe that the pandemic will not affect their willingness for COVID-19 vaccination. The proportion of such rural residents is higher than that of such urban residents. In the age group of 46–60 years old, the proportion of such urban residents is higher than that of such rural residents. Those ≥61 years old both in urban and rural areas maintain a rate of about 3%.

According to the results of the survey, if a new case of COVID-19 occurred in the local border areas, 54.8% of the urban respondents and 59.2% of the rural respondents said their willingness to get vaccinated would be affected; however, the willingness of 39.9% (3527/8849) of the respondents would not be affected. Regarding the population who persisted in such view, the proportion was higher in urban areas than rural areas (42.6% vs. 36.0%) (Fig 2A), especially age from 46 to 60, but before the age of 45, the proportion was higher in rural than urban. With the increase of age, the proportion of people holding various cognitive views decreased, and there were very close (about 3%) when aged ≥61 (Fig 2B).

### Factors influencing willingness to vaccinate against COVID-19

The results of the survey reveal that 27.9% (2473/8849) of the respondents believed that new cases of COVID-19 would significantly affect their willingness to get vaccinated against COVID-19, while the remaining 39.9% (3527/8849) of the respondents believed that new cases would not affect their willingness.

#### A single factor analysis was used to analyze the correlation between new epidemic situation and willingness to vaccinate

The results showed that factors such as residence, age, marital status, occupation, education, basic living security, physical health, awareness of vaccination, willingness to vaccinate, whether to pay for vaccination were associated with willingness to vaccinate against COVID-19 among border residents all *p*<0.25 (Table 4), but the difference between genders was not significant (*OR* = 1.004, *95%CI* 0.905–1.114, *p* = 0.941).

**Table 4:** The impact factors of new COVID-19 epidemic on vaccination willingness with logistic regression analysis (n = 6000).

Influencing factors of new infectious disease affecting COVID-19 vaccination	n	No impact on willingness to get vaccinated%	Single factor analysis *OR 95CI%*	Multivariate logistic analysis *OR 95CI%*
Place of residence:				
Urban	3728	62.10%	0.93*(0.83,1.03)	0.96 (0.84,1.10)
Rural	2272	37.90%	Reference	Reference
Age 18–35 years old	2616	43.60%	1.50***(1.26,1.77)	0.71**(0.58,0.86)
36–55 years old	2684	44.70%	0.97 (0.82,1.15)	1.03(0.86,1.23)
≥56 years old	700	11.70%	Reference	Reference
Marital status: Married	4213	70.20%	0.71*** (0.62,0.80)	1.19**(1.01,1.39)
Divorced	310	5.20%	0.47 ***(0.36,0.61)	1.81***(1.35,2.43)
Widowed	82	1.40%	0.84 (0.54,1.32)	0.97 (0.60,1.57)
Unmarried	1395	23.30%	Reference	Reference
Education				
Secondary school/high school	1248	20.80%	0.82** (0.71,0.95)	1.17(0.98,1.38)
College/Undergraduate	2816	46.90%	1.02 (0.91,1.15)	1.01 (0.83,1.21)
Master’s degree and above	35	0.60%	1.47 (0.75,2.87)	0.67(0.33,1.37)
Junior high school and below	1901	31.70%	Reference	Reference
Occupation:				
Public officials	1192	19.90%	0.84 (0.72,0.98)	1.37*** (1.08,1.73)
Worker/self-employed	812	13.50%	0.82* (0.69,0.98)	1.27** (1.04,1.56)
Medical staff	1337	22.30%	0.96 (0.83,1.11)	1.52*** (1.20,1.91)
Other	1020	17.00%	0.78** (0.67,0.92)	1.51*** (1.23,1.86)
Farmers	1639	27.30%	Reference	Reference
Work/life security situation				
Unstable job but guaranteed income	1716	28.60%	1.10* (0.98,1.24)	1.08 (0.93,1.26)
No fixed-job, basic income guarantee	630	10.50%	0.96 (0.80,1.14)	1.28** (1.04,1.58)
No job, basic life difficulties	183	3.10%	1.13 (0.83,1.52)	1.01 (0.77,1.51)
Students in school, no income	319	5.30%	1.27 **(1.01,1.60)	1.22 (0.94,1.61)
Stable job and wealthy	3152	52.50%	Reference	Reference
Own healthy status				
Not healthy	2030	33.80%	1.21*** (1.09,1.35)	1.13 (0.99,1.28)
Healthy	3970	66.20%	Reference	Reference
There are contraindications to vaccination				
No	4661	77.70%	0.71*** (0.63,0.80)	1.29*** (1.14,1.47)
Yes	1339	22.30%	Reference	Reference
Concerns about virus mutation				
No	5215	86.90%	0.69***(0.60, 0.81)	1.31** (1.12,1.54)
Yes	785	13.10%	Reference	Reference
Concerns about the safety of vaccines				
No	4515	75.30%	0.50*** (0.45,0.57)	1.85*** (1.61,2.11)
Yes	1485	24.80%	Reference	Reference
Do you think the current COVID-19 vaccine can effectively prevent and control the epidemic?				
Definitely can	3453	57.60%	0.60** (0.37, 0.98)	1.58 (0.961.2.62)
Should be able to	2297	38.30%	0.73*(0.45,1.18)	1.55 (0.94,2.57)
Do not know	68	1.10%	0.94 (0.54,1.65)	1.30(0.73,2.31)
Will not be able to	182	3.00%	Reference	Reference
If you need to pay for the COVID-19 vaccine, will it affect your willingness to get vaccinated?				
Whether the vaccine is paid for does not affect my willingness to get vaccinated	4173	69.50%	1.60 ***(1.32,1.95)	0.60*** (0.49,0.73)
Payment may affect my willingness and to get vaccinated	1073	17.90%	3.42***(2.74, 4.27)	0.32 ***(0.25,0.40)
Very worried about the cost and hesitant to get vaccinated	206	3.40%	1.78 **(1.28, 2.49)	0.60*** (0.42,0.85)
No vaccination if payment is needed	548	9.10%	Reference	Reference

*p<0.25,

p**<0.05,

p***<0.01

#### Variable assignment

Taking factors regarding whether the new local cases would have an impact on the willingness to get vaccinated against COVID-19 (Yes = 1, No = 0) as the dependent variable, a single factor analysis was made on the relevant factors that affect the willingness of urban and rural residents to get vaccinated against COVID-19, so as to determine the health status of the respondents, with Yes (healthy) = 1 and No (non-healthy) = 0. The factors affecting vaccination were denoted as follows: whether the vaccine needs to be paid for does not affect my willingness to get vaccinated = 1, whether the vaccine needs to be paid for affects my willingness to get vaccinated = 2, I am very worried about the cost and I am hesitant to get vaccinated = 3, I will not pay for vaccination = 4); whether the COVID-19 vaccine can effectively prevent and control the epidemic: (Definitely can = 1, Should be able to = 2, Do not know = 3, Will not be able to = 4) and (Region: urban = 1, rural = 2; marital status: married = 1, divorced = 2, widowed = 3, unmarried = 4; education: high school / technical secondary school = 1, college/undergraduate = 2, master’s degree and above = 3, junior high school and below = 4; occupation: public official = 1, worker/self-employed = 2, medical staff = 3, others = 4, farmer = 5; work/income situation: unstable job but income is guaranteed = 1, no fixed job, basic income guaranteed = 2, no job, basic living difficulties = 3, school students, no income = 4, stable job, wealthy = 5). The independent variables were included in the multivariate regression model.

The respondents who believed there would be no impact included: those aged 18–35 years old (*OR* = 0.71), and those who were worried about the payment of the COVID-19 vaccine (*OR* = 0.60, 0.32, 0.60). The results reveal that the epidemic situation of COVID-19 had a greater impact on said respondents’ willingness to get vaccinated compared with those who thought that the new COVID-19 epidemic would affect their willingness to get vaccinated (*p*< 0.05). Meanwhile, respondents who were divorced (*OR* = 1.81) and married (*OR* = 1.19),those in an occupation other than farming (OR = 1.37, 1.27, 1.52, 1.51), those with contraindications to vaccination (*OR* = 1.29), and those worried about virus mutation (*OR* = 1.31), and those concerned about the safety of vaccines (*OR* = 1.85), thought that the epidemic situation would not affect their willingness to get vaccinated (all *p* < 0.05) (Table 4).

## Discussion

The prevention and control of epidemics in border areas is a common problem faced by governments of all countries [21]. Maintaining the stability of borders, protecting the lives, property and health of people in border areas, minimizing the import of external infectious diseases, and building a solid border epidemic prevention barrier are essential for ensuring domestic security [22]. The Chinese government has introduced a series of strict border control measures to effectively control imported COVID-19 cases from abroad [23]. Said measures include improving the legal system in border areas, strictly controlling non-essential personnel exchanges between internationals, and limiting large areas to a maximum capacity [24]. The internal gathering and entry and exit of personnel are subject to the approval of declare in advance, people entering high-risk areas need to be isolated and observed at fixed points [25], and grid-based and refined management of communities, villages, and urban needs to be implemented. These effective epidemic prevention and control measures have been approved by the people. Although support and cooperation have played a positive role in effectively curbing the import and spread of infectious diseases [26], long-term persistence requires a large amount of manpower and material resources [27] and will seriously hinder economic and social development. Vaccination is an efficient and economical epidemic prevention and control measure recognized by the international community [28]. However, in the early stage of the COVID-19 vaccine promotion [29], due to doubts about the effectiveness and safety of COVID-19 vaccines, people were hesitant to get vaccinated [30]. As a result, the promotion and implementation of COVID-19 vaccines was considerably difficult, which led to the vaccination rate being low and the speed of vaccination being slow [31]. Therefore, timely popularizing the knowledge of vaccination, actively publicizing the benefits of vaccination in the fight against COVID-19, and alleviating people’s concerns about vaccination in a timely manner may improve people’s willingness to get vaccinated.

The effective prevention and control of infectious diseases in border areas and the fine layout are of considerable strategic significance. Timely completion of COVID-19 vaccinations may be the best way to control the spread of infectious diseases [32]. Once the domestic epidemic is effectively controlled, the key to further epidemic prevention and control is to focus on cases imported from the border [33], but borders have frequent personnel exchanges and active trade, making strict control considerably difficult. The results of the survey reveal that, on the Chinese border region to Vietnam in Yunnan, 94.7% of the respondents in border urban and rural areas believed that the COVID-19 vaccine could effectively prevent and control the spread of the COVID-19 epidemic, and 93.6% border respondents were eager to receive the COVID-19 vaccine as soon as possible. Among the respondents, rural residents had the highest urgency. The reasons may be as follows: (1) The economic foundation and education level of rural border areas are relatively backward. The rural residents [34] have a low educational level, some residents have no fixed income, and a very small number of board residents have difficulties in basic living. To date, there is a lake of specific medicine for COVID-19, and the treatment cost is considerably expensive [35]. Promotion of vaccines can minimize the spread of infectious diseases [36]. However, rural residents have difficulties getting vaccinated due to unstable work or insecure income[37]; (2) Owing to the weak economic foundation of border rural areas, inadequate medical security, lack of regular physical examinations and correct assessment of their own physical health [38], the proportion of rural residents who think they are not healthy is higher than that of urban residents; (3) Yunnan Province is a border region in southwestern China, with mountainous areas accounting for more than 80% of the region, and the relatively backward transportation has severely restricted the economic and social development; and (4) The border between the Prefecture of Honghe and Vietnam is 848 kilometers long [16]. Some borders are only separated by a mountain or a river. Due to difficulties in border control, smuggling occasionally occurs, and epidemics of imported infectious diseases are also prone to occur, meaning that the border has become a high-risk area for imported infectious diseases. As such, comprehensive measures such as actively developing the economy, making up for the shortcomings of poor traffic, consolidating the border air defense system, constantly improving the education and health awareness of border residents, regularly organizing physical examination or health assessment for high-risk industries or elderly people, and enabling them to correctly understand diseases and promote self-health may be of positive significance to strengthen border epidemic prevention and control.

Vaccination against COVID-19 can effectively prevent and control the epidemic of infectious diseases [32], but the safety and effectiveness of the vaccine are the prerequisites for the promotion and popularization of the vaccine. The results of the survey reveal that more than 95% of the respondents believed that the domestic COVID-19 vaccines produced were very safe or relatively safe, and more than 97% of urban and rural residents were willing to take the initiative to promote vaccination after being vaccinated. Factors such as the health status of residents in border areas, whether there were contraindications for vaccination, whether they were worried about virus mutation, whether COVID-19 vaccination was paid for, and other factors affected COVID-19 vaccination among urban and rural residents in border areas (*p*<0.05). Said factors were related to whether there were new COVID-19 cases in the border area. The border epidemic situation would have an impact on the willingness of people of different age groups to get vaccinated against COVID-19, with the greatest impact being on urban and rural residents aged 36–45 years old. Therefore, the government needs to strengthen the popularization of vaccination knowledge [39], such as informing the public through television, radio, online media, propaganda posters why vaccination is needed, what are the indications and contraindications of vaccination, whether payment is required, and the mutation of the virus countermeasures, etc. Residents need to actively cooperate with the government, take the initiative to understand the knowledge related to vaccination [40], actively participate in the prevention and control of the epidemic, and take the initiative to get vaccinated.

China’s epidemic prevention and control and economic recovery have achieved positive results [12, 41]. China has a population of over 1.4 billion. As of August 26, 2021, 1.8 billion doses of the COVID-19 vaccine had been successfully administered [42]. However, neighboring countries such as India and Vietnam have experienced a surge in the number of confirmed COVID-19 cases since entering the second quarter of 2021 [14], which has brought significant challenges to epidemic prevention and control in the southwest border areas of China. The results of the survey reveal that in the border areas between China and Vietnam, 54.8% of urban respondents and 59.2% of rural respondents believed that if new COVID-19 cases occurred in local areas, their willingness to get vaccinated would be affected. The reasons are as follows: (1) the COVID-19 epidemic in China has been effectively controlled, but is still spreading abroad [43], and the uncertainty of the future epidemic situation has increased psychological fluctuations [44];(2) The border is a high-risk area for epidemic import, and border control is complex, with the risk of epidemic outbreak at any time; and (3) the COVID-19 variants and the enhancement of virus infectivity have led to people’s concern about the effectiveness of the vaccine. Thus, to better maintain border stability and maximize the protection of the lives and health of the people on the border, it is necessary to fully understand the current situation of epidemic prevention and control in border areas, identify outstanding problems, and adjust the national border epidemic prevention and control policies in a timely and appropriate manner according to the development trend of the epidemic in neighboring countries in the border area, in addition to the implementation of efficient and precise measures.

The epidemic situation would affect people’s willingness to get vaccinated. The results of the survey reveal that 42.6% of urban respondents and 36.0% of rural respondents said that even if new cases of COVID-19 occurred, their willingness to get vaccinated would not be affected. People aged 18–35, people who were not healthy, and people with concerns about paying for vaccination, may have inadequate understanding of the epidemic situation, inadequate assessment of their own physical condition, and insufficient understanding of the national free vaccination policy, and thus, may believe that a new case would have no impact on their willingness to get vaccinated. People who were divorced, people with relatively stable jobs / guaranteed income, people with contraindications to vaccination and people worried about virus mutation may have higher knowledge literacy. These people have a fuller understanding of the epidemic situation and the advantages and disadvantages of vaccination, and their willingness to get vaccinated is more likely to be affected by the epidemic situation. Therefore, there is an urgent need to improve the cognition level of border residents on epidemic prevention and control, timely publicize the relevant national policies on epidemic prevention and control, actively popularize the knowledge of vaccination, and change the one-sided belief of residents, which may be of positive significance to promote vaccination.

The levels of perception and willingness of COVID-19 vaccination varied by region and by age group [45]. Regarding those who believed that new COVID-19 cases would have no effect on their willingness to get vaccinated, the proportion of such people was higher in urban areas than in rural areas, and the peak age was 36–45 years old. It may be related to better medical and health care conditions in urban areas. At the same time, in the 18–45 years age group, the proportion was higher in rural areas than in urban areas, while in the 46–55 years age group, the proportion was higher in urban areas than in rural areas. For the age group ≥61 years, urban and rural areas were nearly equal (about 3%). Such a trend could be attributed to the fact that before the age of 45, the body is strong and resistant, and most people are busy focusing on their career, meaning that new cases of COVID-19 would have minimal influence on their willingness to get the COVID-19 vaccine, especially in the rural areas of the border, in which the proportion of the population is 28.3%. After the age of 46–55, rural residents in frontier areas may gradually realize their physical deterioration and become aware of health problems, which could affect their ability to combat COVID-19, and thus, gradually realize that not vaccinating for disease prevention is disadvantageous, so they become more willing to get vaccinated. The 46-55-year-old border urban residents are mostly close to retirement age. At this stage, work and life are less stressful, and one can enjoy post-retirement life and medical security, such that they have a higher awareness of the epidemic situation than in rural areas and firmly believe that not being vaccinated has little impact for themselves, but the proportion of people who adhere to this cognitive view remains at a low level (about 10%). For those aged over 61, the body’s resistance is significantly weakened, and various health problems are likely. New cases of COVID-19 would affect the willingness of urban and rural residents to get vaccinated, and the proportion of people who still insist that the epidemic situation would not affect their willingness to get vaccinated was 3%, a relatively low number. Therefore, there is a need to formulate a targeted vaccination plan for urban and rural residents of different ages in different regions to improve their willingness to get vaccinated and accelerate the process of vaccination.

The limitations of this study are as follows: (1) This study is a sample survey, although being careful questionnaire designed, detailed sample size calculation and active mobilization before the survey, some people still cannot fully participate in the survey due to the limitations of infectious diseases, resulting in inevitable sampling bias in the sample survey; (2) To enable the widest range of residents to participate in the survey, the questionnaire fully considers the needs and possible problems of people of different ages and educational levels for filling in the questionnaire. The survey conducted with electronic questionnaire and paper questionnaire is not yet perfect and needs media publicity and participation of local online media; (3) This study has received strongly support from community, township hospitals and health departments, but the investigation is still insufficient due to the limited personnel and funds.

## Conclusions

In the border areas between China and Vietnam, urban and rural residents are more motivated to get a domestic COVID-19 vaccine. Urban and rural residents have different perceptions of COVID-19 vaccination. The epidemic situation affects the willingness of urban and rural residents of different age groups to get vaccinated. There is a need to conduct vaccination related knowledge popularization and health education according to the characteristics of people in different areas and different age groups in border areas, improve their health concepts and change their cognition of adverse epidemic situations, so as to constantly consolidate the epidemic prevention and control work, and continuously promote the popularization of vaccines, thereby facilitating the handling of the changing border epidemic situation and various emerging infectious diseases.

## Supporting information

S1 Fig A) Border rural residents: 95.1% (3461/3639) urgently expecting COVID-19 vaccination, higher than the 92.6% (4826/5210) for urban residents. Especially, 51.3% (1866/3639) of rural residents show the highest demand. B) Those of age ≥ 56 have the strongest willingness, accounting for 52.0% (537/1032).
(TIF)

S2 Fig A) Rural areas: 59.2% (2154/3639) thinks so, higher than the 54.8% (2855/5210) for urban areas. B) Border residents of age ≤45 believe that the pandemic will not affect their willingness for COVID-19 vaccination. The proportion of such rural residents is higher than that of such urban residents. In the age of 46 to 60 years old, the proportion of such urban residents is higher than that of such rural residents. Those ≥ 61 years old both in urban and rural areas maintain a rate of about 3.
(TIF)

S1 File (DOCX)

S2 File Survey questions. (DOCX)

S3 File Survey results. (XLSX)
