# Supplementary figures and images for: Influence of epidemic situation on COVID-19 vaccination between urban and rural residents in China-Vietnam border area: A cross-sectional survey
Source: PLoS One. 2022 Jul 21;17(7):e0270345. doi: 10.1371/journal.pone.0270345 (PMC9302727; doi:10.1371/journal.pone.0270345)

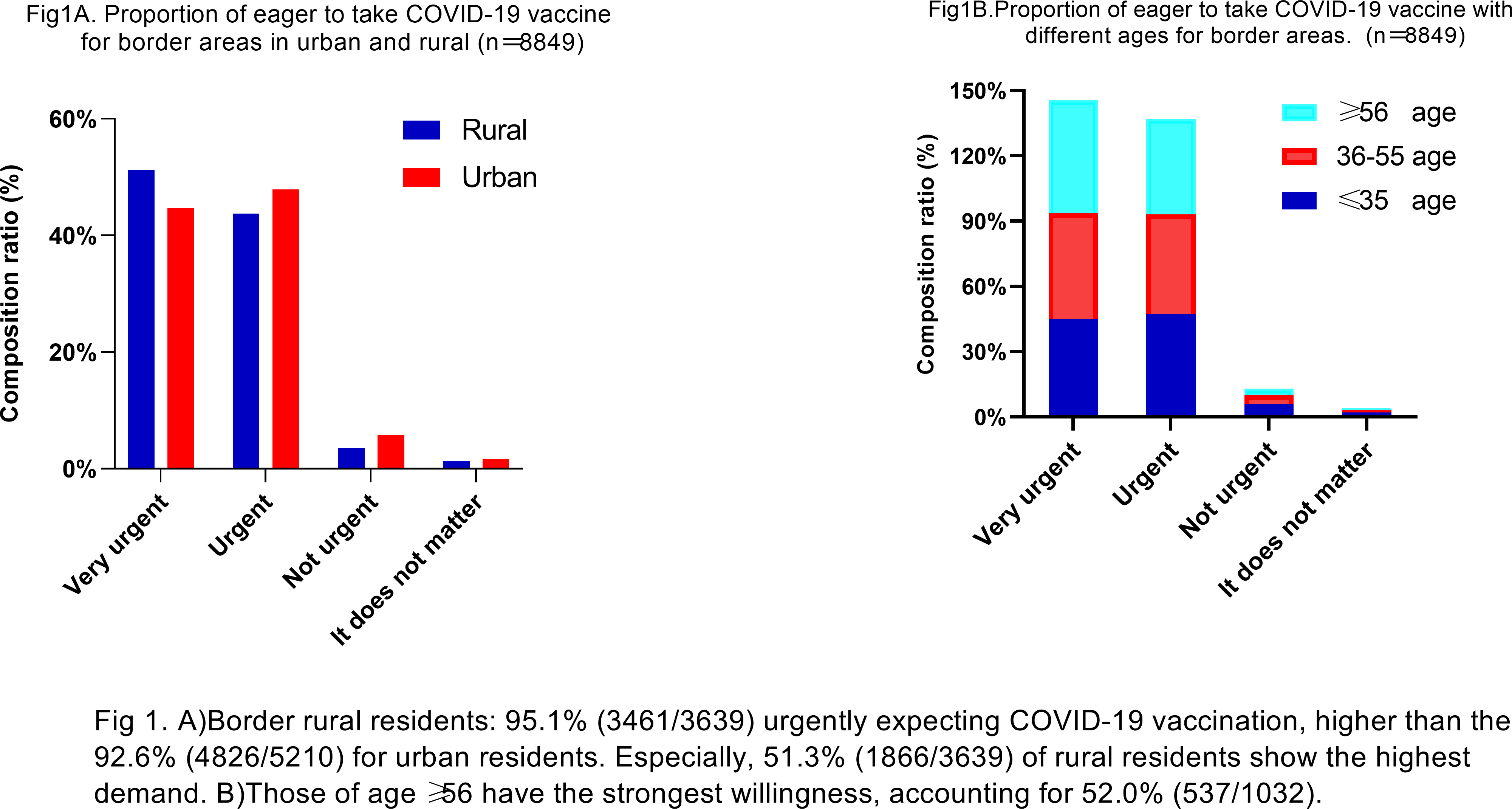

Supplement: S1 Fig — A) Border rural residents: 95.1% (3461/3639) urgently expecting COVID-19 vaccination, higher than the 92.6% (4826/5210) for urban residents. Especially, 51.3% (1866/3639) of rural residents show the highest demand. B) Those of age ≥ 56 have the strongest willingness, accounting for 52.0% (537/1032). (TIF) [file pone.0270345.s001.tif]

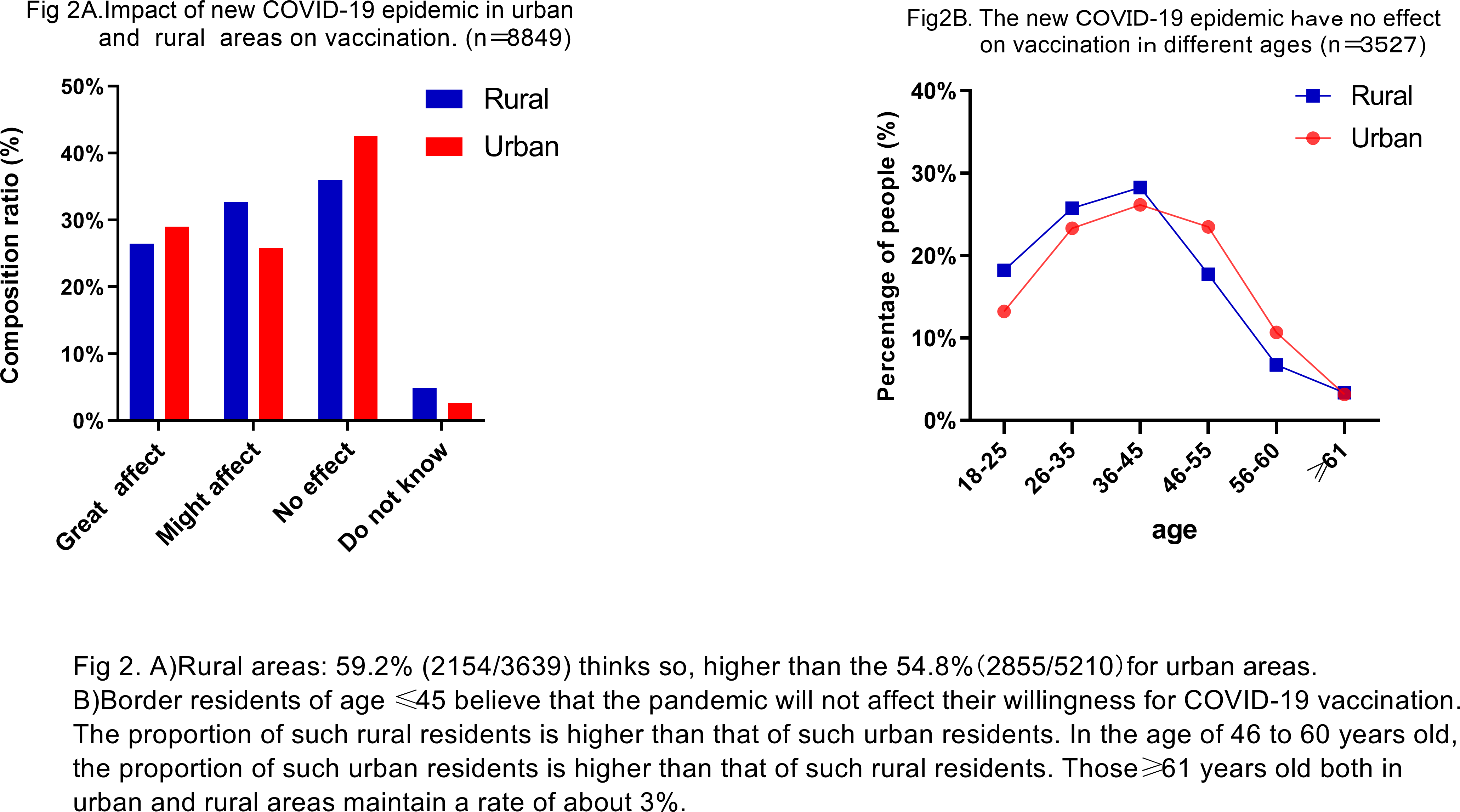

Supplement: S2 Fig — A) Rural areas: 59.2% (2154/3639) thinks so, higher than the 54.8% (2855/5210) for urban areas. B) Border residents of age ≤45 believe that the pandemic will not affect their willingness for COVID-19 vaccination. The proportion of such rural residents is higher than that of such urban residents. In the age of 46 to 60 years old, the proportion of such urban residents is higher than that of such rural residents. Those ≥ 61 years old both in urban and rural areas maintain a rate of about 3. (TIF) [file pone.0270345.s002.tif]
