# Supplementary material for: Influence of epidemic situation on COVID-19 vaccination between urban and rural residents in China-Vietnam border area: A cross-sectional survey
Source: PLoS One. 2022 Jul 21;17(7):e0270345. doi: 10.1371/journal.pone.0270345 (PMC9302727; doi:10.1371/journal.pone.0270345)
Supplement: S1 File — (DOCX) [file pone.0270345.s003.docx]

**新冠肺炎疫情期间居民对接种新冠肺炎疫苗的认知及接种意愿调查**

尊敬的先生/女士您好！

我们非常高兴地希望能邀请您参加本次“**新冠肺炎疫情期间居民对接种新冠肺炎疫苗的认知及接种意愿调查**”，本次问卷调查已经征得昆明医科大学第五附属医院学术伦理委员会的审批同意，您将以匿名方式自愿参与。您填报的信息仅用于数据分析，对您没有任何不利的影响，您可放心做答，感谢您的参与。（注意：您若同意参与本次问卷调查，请在相应的选项上打钩，并依次完成所有的选项，若您不同意/不想继续参与，您可以随时选择退出，退出后您填报的信息将作废）。

**□同意**

**□不同意**

1.**良好的身体是预防接种的基本条件，请您对自己的身体健康状况做一个自我评价(注意，你的自我评价不作为预防接种的依据，因为该工作需要专业的医务人员来评估完成)。（单选）**

| □非常健康（没有急慢性疾病，身体和心理保持健康）  □不健康（亚健康+疾病状态）（亚健康：无慢性基础疾病，但在生理、心理和社会状态上压力很大；疾病状态：有高血压、糖尿病、冠心病等急慢性疾病,或者需要长期服药） |
| --- |
|  |

**2.** **您是通过哪些途径获取接种新冠疫苗信息的？（多选）**

□**电视、广播和其他媒体**；

**□手机、电脑等社交平台**

**□报纸、杂志、布告栏**

**□相互交流/交流学习**

**□其他**

**3.您认为接种新冠疫苗可以有效预防和控制疾病蔓延么？（单选）**

□一定可以；

□应该可以；

□不确定；

□不知道

**4.** **您会第一时间迫切希望能接种到疫苗吗？ （单选）**

□非常迫切；

□比较迫切；

□无所谓；

□不迫切

**5.对于我国免费接种新冠肺炎疫苗，您的接种意愿？（单选）**

□非常期待；

□比较期待；

□随意；

□不期待

**6.您不考虑接种新冠肺炎疫苗的主要原因有哪些? （多选题，您可以选择一个或者多个选项）**

□担心疫苗的安全问题；

□有接种疫苗的禁忌症；

□对病毒变异的担忧；

□其他因素

**7.** **如果您所在地有新的COVID-19疫情发生，会影响您接种疫苗的意愿吗？（单选）**

□一定会影响；

□可能会影响；

□不会影响；

□不知道

**8.您会主动宣传和动员其他人来接种疫苗吗？（单选）**

□是的；

□可能会；

□不会；

□与他人无关

**9.** **据您所知，您认为目前接种的国产新冠肺炎疫苗的安全性如何？（单选）**

□非常安全放心；

□比较安全；

□不太安全；

□特别不放心

**10.** **您是否知道 COVID-19 疫苗需要二次强化接种。 （单选）**

□是的；

□不是

**11. 您认为接种 COVID-19 疫苗可以预防各种肺炎感染吗？ （单选）**

□是的；

□不是；

□不知道

**12.假如新冠肺炎疫苗的接种是需要付费的，会影响您接种疫苗的意愿吗？（单选）**

□是否付费都不影响我接种疫苗的意愿和积极性；

□可能会明显影响我接种疫苗的意愿和积极性；

□非常担心费用问题而犹豫是否继续接种；

□完全没有接种疫苗的费用担忧和影响

**（1）您的性别** ？

□男 □女

**（2）您的年龄？**

□18-25岁 ；

□26岁-35岁；

□36岁-45岁；

□46岁-55岁；

□56-60岁

□＜18岁

□≥61岁

**（3）您的婚姻状况？**

□未婚

□已婚

□离异

□丧偶

**(4)您的工作/生活情况？**

□工作稳定，生活充裕

□工作不稳定但生活充裕

□无固定工作，基本生活有保障

□无工作，基本生活困难

□在校学生，无收入

**（5）您的教育程度？**

□初中及以下；

□中专/高中；

□大学本/专科；

□硕士及以上

**(6)您的职业类别？**

□机关/事业单位公职人员

□工人/个体经营者

□医务人员

□农民

□其他

**（7）您的居住地？**

□城镇 □农村

备注：以上问卷为完整调查问卷的一部分，与本研究无关的内容未展示。
