# Supplementary material for: Influence of epidemic situation on COVID-19 vaccination between urban and rural residents in China-Vietnam border area: A cross-sectional survey
Source: PLoS One. 2022 Jul 21;17(7):e0270345. doi: 10.1371/journal.pone.0270345 (PMC9302727; doi:10.1371/journal.pone.0270345)
Supplement: S2 File — (DOCX) [file pone.0270345.s004.docx]

**Investigation of Cognition and Willingness to Vaccination Against COVID-19**

Dear participant,

We are happy to invite you to participate in this questionnaire survey. Which have reviewed and approved by Ethics Committee. The questionnaire is anonymous and voluntary. The information you fill in the questionnaire will only be used for data analysis, please be assured. Thank you for your participation. (**Note**: If you agree to participate in this survey, please tick the corresponding options and complete all the options in turn. If you **do not agree**, you can choose to leave at any time, and the information you have already filled in will be invalidated after leaving).

**□Agree**

**□Disagree**

**1.A good healthy is the basic condition for vaccination. Please make a self-assessment of your health about yourself. (Single choice)**

**（Notice ：This “self-Assessment of Health Status ”for yourself is not the basis for vaccination, because this evaluation work requires professional medical personnel to complete）**

| □**Yes, Health**（No acute or chronic diseases, physical and mental maintain health）  □**No, Unhealthy**（Sub-Health: no physical disease, but with high pressure in physiological, psychological and social states, and （or） Disease status: With acute and/or chronic diseases such as hypertension, diabetes, coronary heart disease, etc., and need long-term medication |
| --- |

**2. How did you obtain the information on vaccination against COVID-19?** (**Multiple Choice,** **one or more choose)**

□ TV, radio, and other media

□Mobile phone, computer, and other social platforms

□Newspapers, magazines, bulletin boards

□Mutual communication/exchange to learn

□Other

**3.Do you think the vaccination can effectively prevent and control the spread of COVID-19? (Single choice)**

**□**Definitely can

□Should be able to

□Won’t be able to

□Do not know

**4. Are you eager to get the vaccine as soon as possible? (Single choice)**

□Very urgent

□Urgent

□Not urgent

□It does not matter

**5.** **How’s your willingness to get COVID-19 vaccine for free of charge (Single choice)**

□Very urgent

□Urgent

□Not urgent

□It does not matter

**6.** **What’s the main reasons for unwillingness to get vaccinated against COVID-19?** (**Multiple Choice**, **one or more choose**, if you choose means you are agreeing, otherwise, you don’t agree)

□YES, worried about the safety of vaccines.

□YES, don’t understand the contraindications about vaccination.

□YES, Concerns about virus mutation.

□Other factors.

**7. If a new case arise in your area, will it affect your willingness to be vaccinated? (Single choice)**

□Yes

□Maybe

□No

□Do not know

**8.** **Are you Willingness to mobilize others to vaccinate？**

□Yes

□Maybe

□No

□Nothing to do with others

**9. How do you think the Safety of domestic COVID-19 vaccines? (Single choice)**

□Very safe and assured

□Moderately safe

□Not very safe

□Especially worried

**10. Did you know that the COVID-19 vaccine requires a revaccination. (Single choice)**

| □Yes |
| --- |
| □No |

**11.** **Do you think getting the COVID-19 vaccine can prevent all kinds of pneumonia infections? (Single choice)**

□Yes

□No

□Do not know

**12.The impact on vaccination if the COVID-19 vaccine needs to be paid for? (Single choice)**

□Whether the vaccine needs to be paid for or not has no affect with me

□Payment may affect my willingness to get vaccinated

□Very worried about payment and hesitating to get vaccinated

□No vaccination if the vaccine needs to be paid for

**（1）Your gender？(Single choice)**

□Male □Female

**（2）Your age（years）? (Single choice)**

□18-25 years old；

□26-35 years old；

□36-45 years old；

□46-55 years old

□56-60 years old

□<18 years old；

□≥61 years old

**（3）Your marital status？(Single choice)**

□Unmarried

□Married

□Divorced

□Widowed

**（4）Your Work/life security situation？(Single choice)**

□Stable job and wealthy

□Unstable job but guaranteed income

□No fixed-job, basic income guarantee

□No job, basic life difficulties

□Students in school, no income

**（5）Your education level? (Single choice)**

□Primary school or below

□Secondary/high school degree

□College/Undergraduate

□Master's degree or above

**（6）Your Occupation? (Single choice)**

□Public officials

□Worker/self-employed

□Medical staff

□Farmer

□Other

**（7）Where you live? (Single choice)**

□Urban

□Rural
